# Music to My Senses: Functional Magnetic Resonance Imaging Evidence of Music Analgesia Across Connectivity Networks Spanning the Brain and Brainstem

**DOI:** 10.3389/fpain.2022.878258

**Published:** 2022-05-19

**Authors:** Jocelyn M. Powers, Gabriela Ioachim, Patrick W. Stroman

**Affiliations:** ^1^Centre for Neuroscience Studies, Queen's University, Kingston, ON, Canada; ^2^Department of Biomedical and Molecular Sciences, Queen's University, Kingston, ON, Canada; ^3^Department of Physics, Queen's University, Kingston, ON, Canada

**Keywords:** functional MRI, human neuroimaging, music analgesia, pain, cognitive/affective pain modulation, network connectivity, structural equation modelling

## Abstract

Pain is often viewed and studied as an isolated perception. However, cognition, emotion, salience effects, and autonomic and sensory input are all integrated to create a comprehensive experience. Music-induced analgesia has been used for thousands of years, with moderate behavioural effects on pain perception, yet the neural mechanisms remain ambiguous. The purpose of this study was to investigate the effects of music analgesia through individual ratings of pain, and changes in connectivity across a network of regions spanning the brain and brainstem that are involved in limbic, paralimbic, autonomic, cognitive, and sensory domains. This is the first study of its kind to assess the effects of music analgesia using complex network analyses in the human brain and brainstem. Functional MRI data were collected from 20 healthy men and women with concurrent presentation of noxious stimulation and music, in addition to control runs without music. Ratings of peak pain intensity and unpleasantness were collected for each run and were analysed in relation to the functional data. We found that music alters connectivity across these neural networks between regions such as the insula, thalamus, hypothalamus, amygdala and hippocampus (among others), and is impacted by individual pain sensitivity. While these differences are important for how we understand pain and analgesia, it is essential to note that these effects are variable across participants and provide moderate pain relief at best. Therefore, a therapeutic strategy involving music should use it as an adjunct to pain management in combination with healthy lifestyle changes and/or pharmaceutical intervention.

## Introduction

Music has been used to alter our perception of pain for thousands of years in cultural, experimental, and clinical environments (1–3). A number of prior studies have demonstrated behavioural effects of music on subjective ratings of pain, including significant decreases in both pain intensity (1, 4–9) and unpleasantness (4, 6, 8, 10, 11), with a 70% higher likelihood of reduced pain (1) and increased pain thresholds (12–14). Furthermore, there is also evidence that the capacity of music to modulate pain is reduced when individuals exhibit higher levels of pain catastrophizing (12). However, a recent meta-analysis found that these effects are highly variable across individuals and studies due to a number of factors including methodological variations across studies, and the underlying mechanisms remain unclear (15).

Hypotheses regarding the underlying mechanisms of music analgesia range from purely distraction (cognition) (16, 17) to purely emotional (valence, arousal, reward) (6, 10, 18). However, these effects may not be separable (19, 20) and an interaction between cognitive, emotional, and sensory domains is the most likely foundation for pain relief from music (3, 6, 10). Lunde et al. (3) described a set of integrated factors, adapted from Tracey and Mantyh (21), which contribute to music analgesia including context, cognition, emotion, neurotransmitters, and predictability of the music itself. A subsequent meta-analysis expanded on this theory by arguing that music can suppress pain by acting as a reward, stress reliever, mood regulator, and distractor (2). This idea is supported by the observation that pleasurable music reduces anxiety and stress through downregulation of the autonomic nervous system (22–24), increasing dopamine and serotonin release in the striatum (12, 25, 26), increasing μ-opioid receptor and endorphin production (27), and recruiting reward and limbic regions to modulate motivation, learning and valuation (18, 25, 28). Anxiety, stress, learning, and reward play prominent roles in how we evaluate the relative importance of painful stimuli and our ability to cognitively and emotionally regulate pain (29–33). Furthermore, increased opioid receptor, endorphin, dopamine, and serotonin production directly interact with the descending opioidergic analgesic pathway consisting of the periaqueductal grey (PAG)-rostral ventromedial medulla (RVM)-spinal cord (34–36). Along with other types of emotional stimuli, music has widely been thought to influence pain via this pathway (3, 13, 27, 37, 38).

While evidence for music analgesia has been described behaviorally, few functional investigations of neural effects in humans have been reported. These include only four previous fMRI studies (8, 13, 37, 39), a study employing EEG (10), and one EMG study (40). Previous fMRI studies have reported attenuation of the anterior cingulate cortex with music during pain stimulation (13), altered resting-state connectivity after music listening in participants with fibromyalgia (8), and differences between pain-plus-music and pain-only conditions across several cortical, limbic, brainstem and spinal cord regions (37).

The objective of this study was to use functional MRI to build on the foundation of existing behavioural evidence to further investigate the neural basis of music analgesia in human participants. We acquired data from healthy individuals during the application of acute noxious thermal stimulation with and without concurrent presentation of pleasurable music individually selected by each participant. Behavioural ratings of pain intensity and unpleasantness were recorded to assess the subjective effects of music analgesia, along with the temperatures required to produce moderate pain. We hypothesised that having a participant listen to pleasant music of their choice while they experience acute heat pain would result in altered descending pain regulation via the PAG-RVM pathway, compared to experiencing the pain stimulus without music. Moreover, we hypothesised that this regulation would be mediated by input from limbic, paralimbic, and reward regions.

## Materials and Methods

All procedures were approved by the institutional human research ethics review board and complied with the Tri-Council Policy Statement on Ethical Conduct for Research Involving Humans. Informed consent for all study procedures was obtained in writing prior to the onset of study training and participants were informed that they could cease participation at any time.

### Participants

Twenty healthy participants (10 female, 10 male) ranging from 21 to 33 years of age (23 ± 3 years, mean ± standard deviation) were recruited from the local community through online advertisements and posted notices. Participants were free of any history of neurological disease or injury, major medical illness, psychiatric disorder or pre-existing pain condition and were not taking any centrally acting medications (i.e., antidepressants) or prescription medication for pain relief. Participants were also instructed to refrain from taking over the counter pain medication (e.g., ibuprofen) on the day of study participation to avoid interference with normal, healthy pain responses.They were also free of any contraindications for the MRI environment including pregnancy, claustrophobia, metal implants or injuries from metal fragments, or inability to lie still. All participants were screened for eligibility through a secure online form.

Eligible participants were asked to complete a battery of validated questionnaires to characterise individual traits of mental health, social behaviours, and pain catastrophizing, which all relate to the sensory and affective dimensions of pain. The questionnaires included the Beck Depression Inventory-II (BDI-II) (41), the State/Trait Anxiety Inventory (STAI) (42), the Social-Desirability Scale (SDS) (43), and the Pain Catastrophizing Scale (PCS) (44). The BDI-II assesses the affective, motivational, cognitive, and somatic symptoms of depression. The STAI measures the transient condition of state anxiety as well as the chronic condition of trait anxiety. The SDS provides an assessment of whether participants are concerned with social approval, such as providing pain ratings in a way that they believe the researchers would approve of. The PCS reflects how individuals respond to pain, such as tendencies to feel helpless and/or magnify the threat value of a stimulus. Participants were not excluded from participating given high or low scores on any of these questionnaires. The resulting scores were used in correlational analyses with functional MRI data to determine if behavioural and psychological traits relate to neurological activity during the experience of pain. Group means for each scale were computed and individual scores were compared with subsequent pain ratings from each participant.

All participants were instructed to bring six selections of familiar, pleasurable music of any genre on a USB-drive in .mp3 format, as music chosen by the participants has been shown to have greater effect than music chosen by the researchers (5, 45). These selections were required to be at least 210 s long to correspond with the length of each scan and yield a rating of 7.5 or higher on 10-point scales of happiness, familiarity, and alertness. During functional scans, participants experienced two experimental conditions: noxious thermal stimulation with simultaneous presentation of pleasurable music (i.e., “Music” runs), and noxious thermal stimulation alone (i.e., “No-Music” runs). Half of the scans were carried out in each condition, in a randomised order. The researchers randomly assigned music selections to the Music runs, and a different selection was played for each music run.

### Experimental Procedures

#### Protocol Training Session

**Figure 1:**
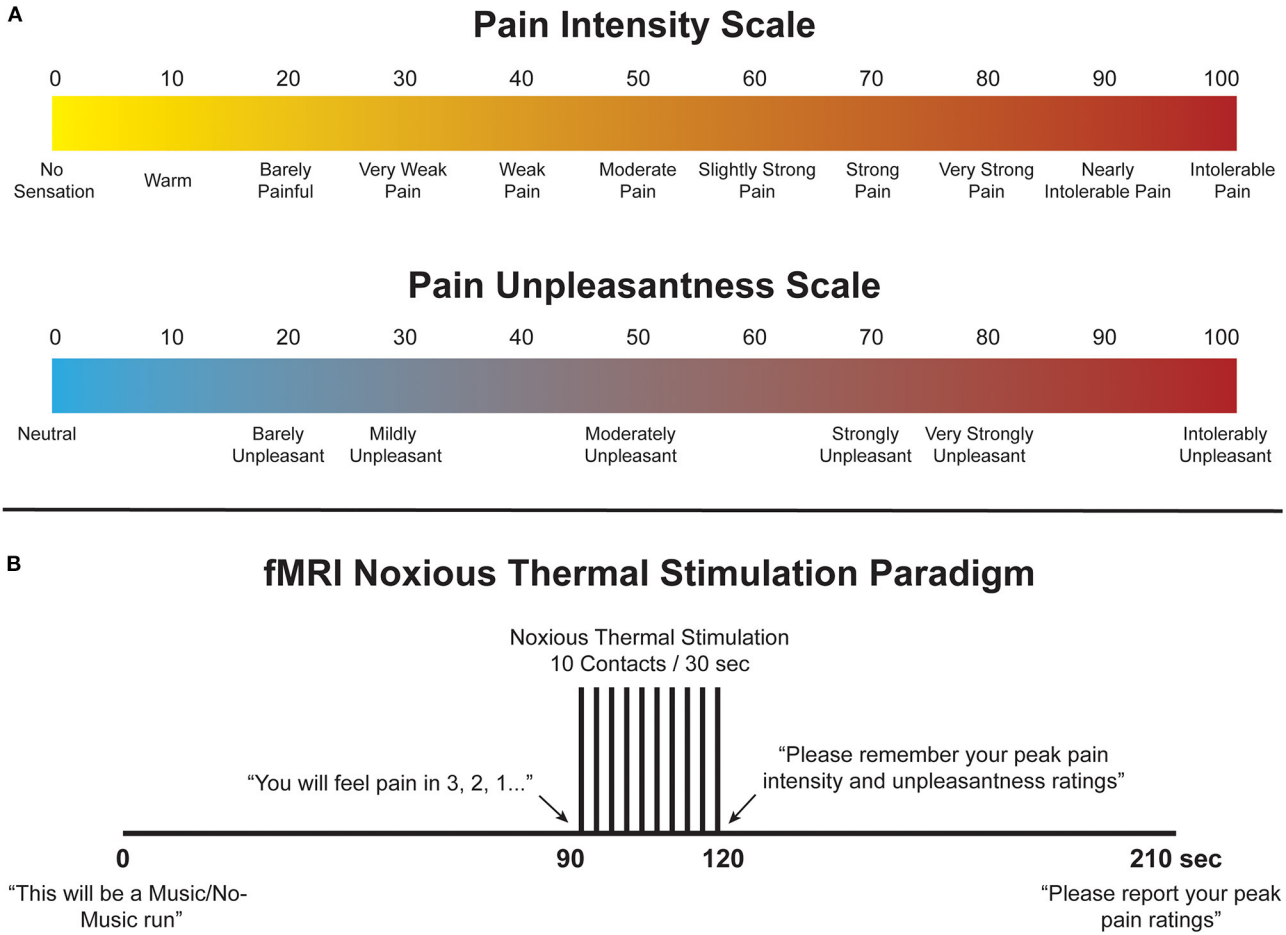
**(A)** Numerical Pain Scales (NPS) used to train participants to rate their pain intensity and unpleasantness. These scales were also displayed during functional scans to aid participants in rating their pain during the experiment. **(B)** Stimulation paradigm used during the training and imaging sessions. For Music runs, the music was synchronised to begin with the onset of scanning and continued throughout until completion.

Immediately prior to imaging, participants underwent a 45-min training session in a “sham” MRI lab within the Queen's University MRI Facility. The purpose of training was to familiarise participants with the study paradigm, including scales with which they would rate their pain experience, the noxious thermal stimulus and timing of stimulation. Participants were trained to use validated 100-point numerical pain intensity and unpleasantness rating scales (NPS), with verbal descriptors at intervals of 10 (Figure 1A) (46–48). Participants were encouraged to rate in increments of 5, and the researcher checked each rating with the participant to ensure that they were becoming familiarised with the scales. They were informed that pain intensity describes more of the sensory/discriminative dimension of pain whereas unpleasantness describes the emotional/affective component of the perceived pain. The ratio of each participants' pain rating to the temperature used to elicit that pain rating was used as a “normalised pain score.” A higher pain score may indicate that participants who experienced a particular pain rating at a lower temperature are more sensitive to pain than those that experienced the same pain rating but required a higher temperature to produce that pain. This method was used to standardise our pain measures given that participants were not all subjected to the same stimulus temperatures.

To elicit acute experimental pain, thermal stimulation was applied with an MRI-compatible robotic contact-heat thermal stimulator (RTS-1), which raised and lowered a 3 cm-square aluminium thermode to and from the participants' skin via pneumatic pistons (49–52). The stimulus was applied to the thenar eminence of the right hand, corresponding to the sixth cervical segment of the spinal cord. The timing and duration of heat-contacts, along with thermode temperature, were under precise control by custom-made software in MATLAB? (Mathworks Inc., Natick, MA). Each test consisted of ten 1.5-s heat contacts over the span of 30 s in order to elicit sustained behavioural and neural responses and to avoid habituation of nociceptors in the skin. Participants were trained with a standard set of temperatures, ranging from 45 to 52 °C presented in the same order, and were individually calibrated to a temperature corresponding to a tolerable average pain rating of 50 intensity units (“Moderate Pain”, Figure 1A) (53). Participants were kept blinded to this objective, as well as to the temperatures used during the tests, to avoid any potential response bias, and the upper limit of 52°C was set to avoid causing damage to the skin. Additionally, participants were instructed to remove their hand from the stimulator if their pain ratings ever exceeded 70 NPS units to avoid causing distress or very strong pain. Once calibrated, participants moved on to the next stage of training.

A mock-up of the MRI scanner (sham-MRI) was used to train participants on the stimulation paradigm and timing that they would experience in the MRI, and to familiarise them with the confined environment. This process was also intended to reduce variations in the data that may be caused by anxiety and bulk motion across repeated fMRI acquisitions. Participants were positioned supine in the sham-MRI with a mirror over their eyes to view a rear projection screen displaying the pain intensity and unpleasantness scales, and the RTS-1 under their right hand. A simulated version of the fMRI protocol was carried out at the calibrated temperature, with recorded MRI sounds played for them on a speaker to simulate the scanner environment. The 210-s stimulation paradigm is shown in Figure 1B. Participants were instructed to silently rate the intensity and unpleasantness of each contact as they felt them, and to remember only the highest ratings on both scales. The peak ratings of pain intensity and unpleasantness were recorded, and the calibration temperature was confirmed or adjusted based on these ratings; this temperature and stimulation paradigm was then used during the subsequent imaging session.

#### Functional MRI Data Acquisition

Functional MRI was carried out on a Siemens 3 tesla MRI system (Siemens Magnetom Trio, Erlangen, Germany). Participants were positioned head-first and supine with foam supports under their knees and arms to minimise bulk motion during scanning. The RTS-1 was positioned at their side, under the palm of the right hand and foam earbuds were provided to ensure optimal sound quality for the music. A 32-channel head coil was used to obtain images of the brain and brainstem and a mirror positioned above the participants' eyes allowed them to view a rear projection screen which displayed prompts for timing of the stimulation paradigm and the pain rating scales during each run. The peripheral pulse was recorded from all participants with a sensor attached to their left index finger, and participants were provided with a squeeze-ball to signal the experimenter in the event of an emergency, or if they did not wish to continue the study. After setup, participants were instructed to remain as still as possible and wait for audio instructions provided to them through the earbuds. Sound quality was checked after the first music run to ensure that participants could hear the music at an appropriate volume over the sounds of the scanner.

Localizer images were acquired in three planes to provide a reference for subsequent slice positioning. A sagittal, T1-weighted anatomical scan was also acquired using a 3D MPRAGE sequence to aid in normalisation of functional data with 1 ×1 ×1 mm^3^ resolution, a repetition time (TR) of 1,760 ms, echo time (TE) of 2.2 ms, inversion time of 900 ms, and flip angle = 9°. In order to produce high quality images of the brain, and maintain this quality in the brainstem, simultaneous multi-slice imaging with an acceleration factor of 2 was used for BOLD functional scans. A gradient-echo imaging method, with echo-planar spatial encoding (GE-EPI), was used with a flip angle of 84°. The 3D volume spanned from the top of the first cervical vertebra to the corpus callosum, with a TE of 35 ms for optimal ${\text{T}}_{2}^{*}$-weighted BOLD sensitivity in the brain. The TR was set at 2,000 ms per volume, and 105 volumes were recorded to produce a time-series spanning 210 s (3.5 min). Data were acquired in 48 contiguous axial slices, 2.1 mm thick, with a 180 ×180 mm field of view, and an 84 ×84 matrix, resulting in 2.1 mm isotropic resolution, with an anterior/posterior phase-encoding direction.

Multiple runs of each condition (Music and No-Music) were acquired in a randomly interleaved order and participants were informed of which condition to expect at the beginning of each run. The stimulation paradigm followed the same timing as in the sham-MRI run (Figure 1B), with periods of expectation, stimulation, and relief. Participants provided their peak pain intensity and unpleasantness ratings at the end of each run, and these ratings were recorded. During the Music condition, the music was synchronised to begin at the exact same time as scanning, and it played throughout the scan. The initial baseline period therefore allowed the participant to become engrossed in the music before the onset of thermal stimulation. In between each run, the MRI operator confirmed that the participant was comfortable and alert before continuing. In total, 10 runs were acquired for each participant, half spent in each condition, in a randomised order.

### Data Analysis

#### Behavioural Analyses

As they were not normally distributed, pain intensity and unpleasantness ratings were investigated across study conditions using 2-tailed, Wilcoxon signed-rank tests, with a significance threshold of *p* < 0.05. The relationships between questionnaire scores, pain intensity and unpleasantness ratings, and normalised pain scores in the No-Music (unmodulated) condition were also tested across all individuals using Spearman's rho correlations, also with significance inferred at a threshold of *p* < 0.05. This was done to determine whether a relationship could be found between participants' individual characteristics and their subjective pain behaviours in an acute, experimental setting.

#### Data Pre-processing

Functional MRI data were pre-processed using Statistical Parametric Mapping software (SPM-12, The Wellcome Centre for Human Neuroimaging, UCL Queen Square Institute of Neurology, London, UK) in MATLAB (MathWorks, Natick, MA, USA). Pre-processing steps included conversion to NIfTI format, co-alignment to correct for bulk motion, and spatial normalisation to pre-defined anatomical templates from the Montreal Neurological Institute (MNI). Images were re-sized to 2 mm cubic voxels prior to normalisation for compatibility with the MNI template, and data were cleaned to reduce noise by fitting and subtracting signal variations corresponding to the motion parameters determined during co-alignment.

**Figure 2:**
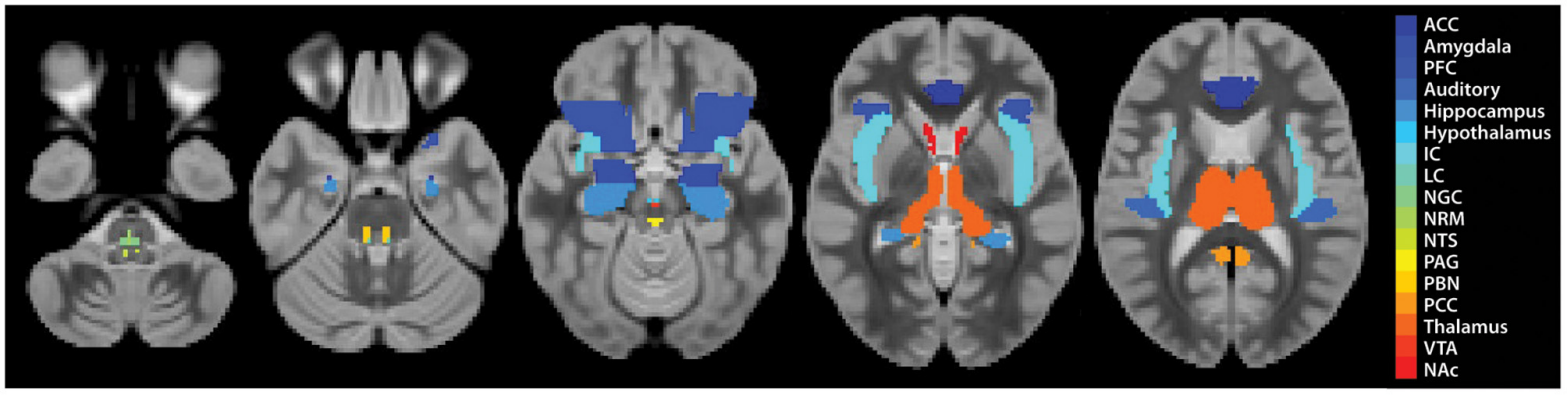
Region definitions for each ROI. Each region is shown as a single colour, as described in the legend.

Subsequent data analyses focused on characterising temporal BOLD responses and relationships between regions known or suspected to be involved in pain, music and emotion processing, and autonomic regulation (54–57) (Figure 2). We aimed to identify the relationships between study conditions (Music vs. No-Music), individual pain scores, the period of the stimulation paradigm (i.e., before, during, and after the noxious stimulus was applied), and personal characteristics (questionnaire scores). For the purposes of prior studies we had created a combined anatomical template and anatomical region map that spans the brain, brainstem and spinal cord (51, 58). For this study, the relevant reference images consisted of the MNI152 template, included in SPM-12, and anatomical regions maps from the CONN15e software (59). Brainstem regions not included in the CONN15e region map were supplemented based on examples and anatomical descriptions (54, 60–64), and freely shared atlases as described by Pauli et al. (65), Keren et al. (66), and Harvard atlases (https://www.med.harvard.edu/AANLIB/).

#### Structural Equation Modelling

**Figure 3:**
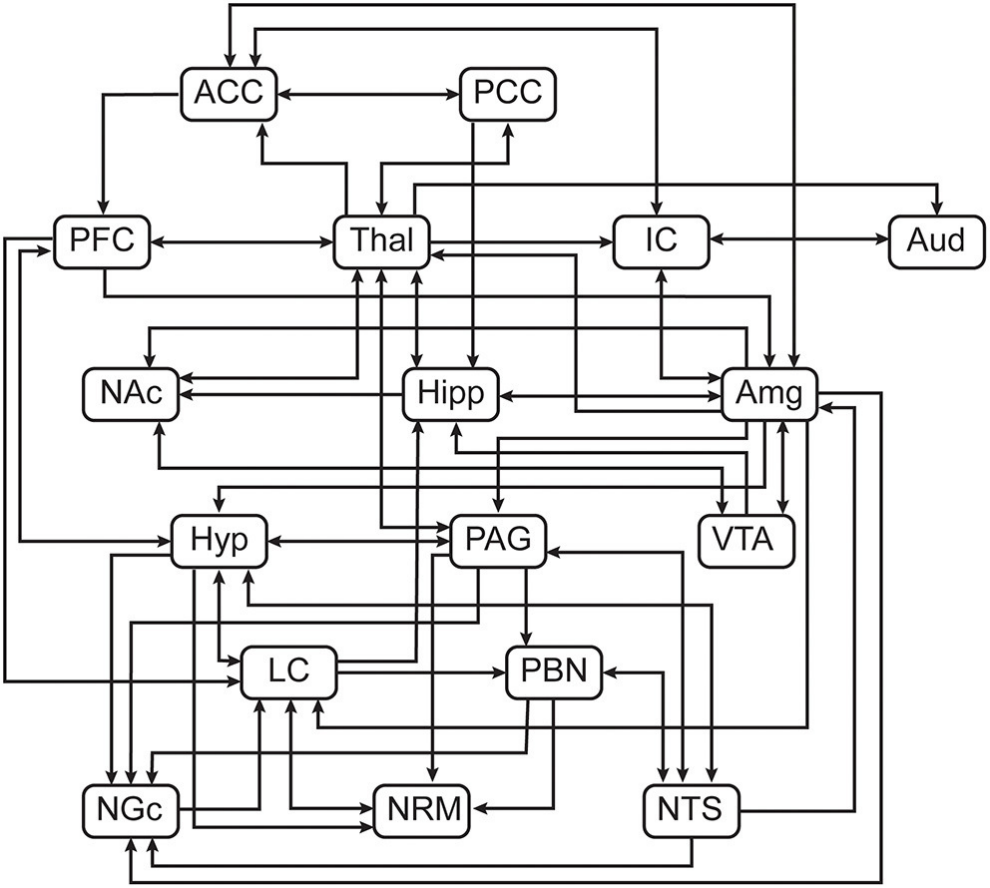
Pre-defined anatomical model of connections between regions of interest.

Structural equation modelling (SEM) is a data-driven family of statistical techniques which are used to identify patterns of correlation/covariance among a set of BOLD responses within and across regions of interest (ROIs) (58, 67, 68). Our SEM methods are focused on characterising temporal relationships by explaining as much variance as possible through use of a pre-defined anatomical model of connections across the brain and brainstem. This pre-defined model is based on known neuroanatomy, including directionality, between ROIs (Figure 3) and includes: ***brain regions***-pre-frontal cortex (PFC), anterior cingulate cortex (ACC), posterior cingulate cortex (PCC), insular cortex (IC), auditory cortex (Aud), thalamus (Thal), amygdala (Amg), hippocampus (Hipp), nucleus accumbens (NAc), and hypothalamus (Hyp); ***midbrain regions***-periaqueductal grey matter (PAG), and ventral tegmental area (VTA); ***pontine regions***-locus coeruleus (LC), and parabrachial nucleus (PBN); ***rostral*** ***medulla regions***-nucleus raphe magnus (NRM), nucleus gigantocellularis (NGc) and nucleus tractus solitarius (NTS) (54). These areas were chosen to cover a comprehensive array of centres for somatosensation, audition, pain processing and perception, music and emotion processing, and autonomic homeostatic regulation (28, 29, 54, 56, 69–71). Some existing anatomical connections were pruned from the network model in order to limit the number of comparisons and to highlight important connections, keeping the focus on connections known to be involved in pain processing and modulation. Data were averaged across clusters of voxels to reduce the number of statistical comparisons to be made and to increase the signal-to-noise ratio over that of single-voxel analyses. Each ROI was functionally divided into 7 sub-regions based on time-series characteristics using k-means clustering. Once defined, identical sub-regions were used across the group for both study conditions. The VTA, however, was divided into 4 sub-regions as it contained fewer voxels than other regions. This process limits potential bias when dividing each ROI into sub-regions as it assumes that each ROI can have more than one function (72–76). Here, we used SEM as a means to investigate coordination across networks of regions. This method has successfully identified robust networks of connectivity across the brain, brainstem, and spinal cord in our previously published work (51, 58, 68, 73–75, 77, 78).

SEM was carried out by means of a general linear model to calculate linear weighting factors (β) which indicate the relative contribution of each connection to the overall network model, using the time-series data across participants, separately for each condition. The calculations are dependent on the following logic: if region A receives input from regions B and C, and the BOLD signal time-series responses in these regions are *S*_*A*_, *S*_*B*_, and *S*_*C*_, respectively, then: *S*_*A*_ = β_*AB*_ *S*_*B*_ + β_*AB*_ *S*_*B*_ + *e*_*A*_; where *e*_*A*_ is the residual signal variation that is not explained by the fit (67). The weighting factors were calculated separately for each network component, consisting of a sub-region receiving input (target) with multiple regions providing input (sources). Networks were investigated for every combination of anatomical sub-regions of each ROI to identify the sub-regions that resulted in the best fits to the data measured.

The significance of connectivity values (β) was determined based on their average values across the group, and the estimated standard errors. Significance was inferred at a family-wise-error corrected *p*_fwe_ < 0.05 which accounted for the total number of network combinations that were tested across combinations of anatomical sub-regions. With this process, connections with β-values which were significantly different than zero were identified and used for subsequent second-level analyses.

#### Analysis of Variance and Covariance

Analyses of variance (ANOVA) and covariance (ANCOVA) were employed as a means of comparing study conditions, time periods of the stimulation paradigm, and behavioural ratings of pain. Connectivity values (β) were used as the dependent variable, with study “Condition” used as one discreet independent variable (Music or No-Music), and the time period (before or during pain, to test an effect of “Stimulation”) as the second discrete independent variables for the ANOVA (i.e. Condition X Stimulation). An ANCOVA was also applied to β-values as the dependent variable, with study “Condition” as a discrete independent variable and “Pain Score” as a continuous independent variable for all 3 time periods of the study paradigm (i.e., Condition X Pain Score, before, during and after pain). Significance of these analyses was inferred at a false discovery rate (FDR) controlled *p* < 0.05.

#### Bayesian Regression

To further investigate temporal details of BOLD responses across ROIs, a Bayesian regression technique was applied to characterise variations across participants in relation to pain unpleasantness ratings and the stimulation temperature. This analysis was used to identify consistent features of BOLD responses in specific regions which were dependent on individual pain behaviours.

Bayesian regression was applied to each point in the BOLD time-series responses in each sub-region, for each individual, using pain unpleasantness ratings and stimulation temperatures as independent variables. The pain ratings and temperatures were first centred so the average values across all participants were equal to zero and scaled so that the largest differences from the average were equal to one. The data were then fit to approximate the consistent BOLD responses (S) at the average pain and temperature ratings (S_0_), plus linear estimates of the BOLD variations with pain ratings (S_p_) and temperature (S_t_) (79): S = S_0_ + pain rating S_p_ + temperature S_t_. The fitting process therefore enables us to estimate BOLD response patterns (S_0_) independent of individual differences in pain sensitivity or the stimulation temperature used, as well as to identify how the BOLD responses varied systematically across participants with different pain responses. The expected BOLD response for a region can thus be identified at the average stimulation temperature, as being S_0_ + S_p_ at the highest pain rating, and S_0_ - S_p_ at the lowest pain rating.

## Results

### Behavioural Results

**Figure 4:**
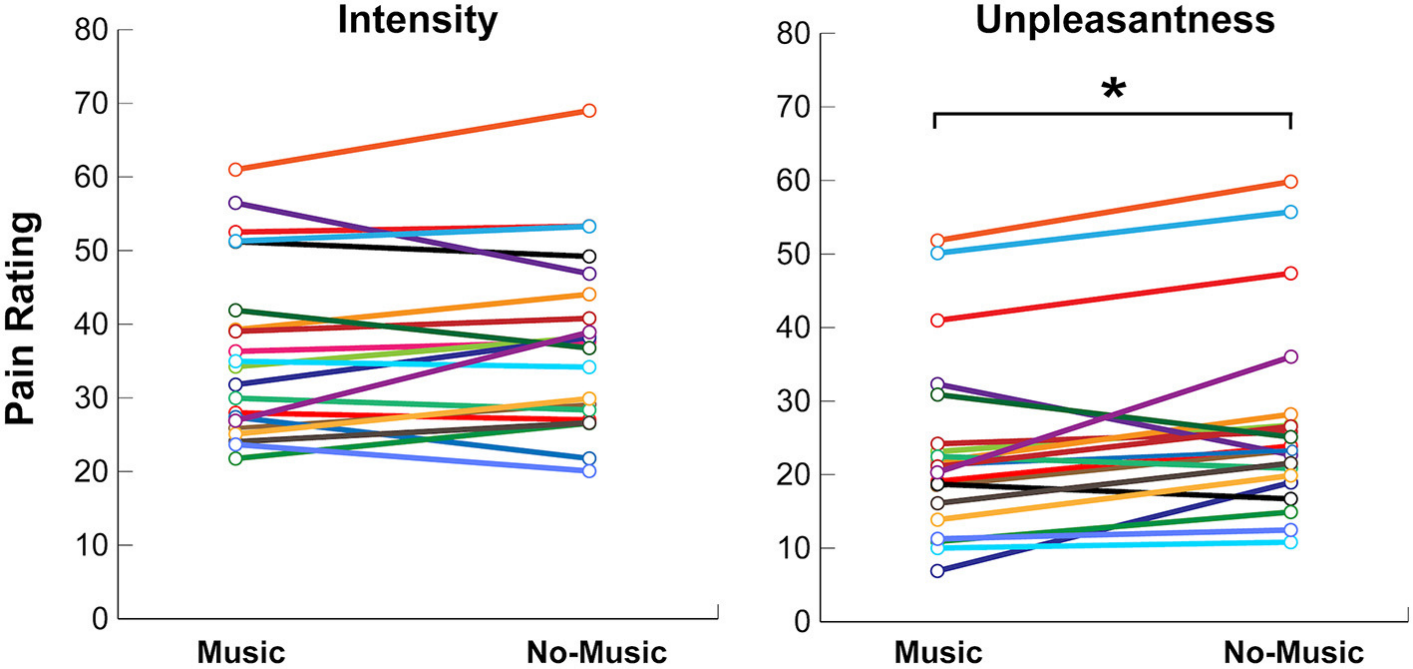
Self-reported behavioural ratings for pain intensity and unpleasantness on numerical rating scales (NPS) during the Music and No-Music conditions. Each coloured line indicates a single participant. Significance at *p* < 0.05 is indicated (*).

Participants experienced a significant reduction in pain unpleasantness during the Music condition as compared to the No-Music condition, when the same temperature was applied. Pain unpleasantness ratings decreased by 13.8% from an average of 26.8 ± 13.4 to 23.1 ± 12.5 [mean ± standard deviation, *Z*_(19)_ = −2.4, *p* < 0.017], between the No-Music and Music conditions, respectively. Pain intensity ratings only decreased by 3.5% from 37.6 ± 12.4 (No-Music) to 36.3 ± 12.2 (Music), however this trend was not found to be significant across conditions (*Z*_(19)_ = −1.10, *p* < 0.27). Some degree of inter-subject variability was noted across participants within each condition, however a consistent trend of lowered pain ratings was observed during the Music condition (Figure 4).

#### Results of the Questionnaires to Assess Participant Characteristics

Group averages indicated that participants scored within normal ranges for all questionnaires including the STAI, SDS, BDI, and PCS (Table 1). Relationships between pain intensity, unpleasantness, normalised pain scores (pain unpleasantness/stimulation temperature), and questionnaire scores were investigated across the group using Spearman's rho correlations. Only two significant correlations were found between pain unpleasantness and BDI (*rho* (18) = −0.49, *p* < 0.028) and normalised pain score and BDI scores (*rho* (18) = −0.49, *p* < 0.03).

**Table 1:** Results of questionnaires to characterise participants' individual characteristics and correlations with pain ratings and normalised pain scores in the No-Music condition.

	**Questionnaire**	**Avg. Score ±SD**	**Percentile/ Range**	**Intensity (*rho*)**	**Unpleasantness (*rho*)**	**Norm. Pain Score (*rho*)**
STAI	State	31 ± 8	37%	−0.23	−0.36	−0.36
	Trait	34 ± 10	45%	0.04	0.03	0.33
SDS		16 ± 5	Average	−0.11	0.26	0.01
BDI		6 ± 7	Average	−0.20	−0.49*	−0.49*
PCS	Total	9 ± 7	22%	0.10	0.08	0.09
	Rumination	4 ± 3	23%	0.23	0.13	0.14
	Magnification	3 ± 2	47%	0.02	0.04	0.05
	Helplessness	2 ± 3	20%	0.01	0.03	0.03

STAI, State/Trait Anxiety Inventory; SDS, Social Desirability Scale; BDI, Beck Depression Inventory; and PCS, Pain Catastrophizing Scale with sub-domains of Rumination, Magnification and Helplessness. Average values and percentiles within normal distributions are indicated where available, or the assessment range is indicated. Correlation rho-values between questionnaire scores and each of pain intensity, unpleasantness and normalised pain scores are also listed, with significant values indicated with an asterisk (*) and df = 18 for all comparisons.

## Functional MRI Results

### Structural Equation Modelling

Significant connectivity was found within the network model during both study conditions, across all periods of the stimulation paradigm (i.e., before, during, and after noxious stimulation). Connections with weighting factors (β) significantly different than zero were observed across brain and brainstem regions and were mainly clustered at the level of the brain (PFC, ACC, PCC, insula, auditory cortex, thalamus, hippocampus, amygdala, NAc), with some projections to and from midbrain regions (PAG, VTA).

### Analyses of Variance and Covariance

The results of the SEM analysis were used in secondary analyses to characterise the relationship between music, pain processing, timing of stimulation, and individual pain behaviours (normalised pain scores). An ANOVA (Condition X Stimulation) was implemented to observe the effect of music on pain processing in relation to the period of the stimulation paradigm. The results demonstrate significant main effects of Condition (Music vs. No-Music) and Stimulation (Before vs. During), in addition to one significant Interaction effect (Table 2). The main effect of stimulation was dominated by connections between the thalamus and insula, primarily from thalamus sub-region 4. Other connections impacted by the shift from before to during noxious stimulation (Time) include the following: amygdala → hippocampus, ACC → insula, insula → auditory cortex, and insula → amygdala. One connection was identified which was impacted by the study condition from the NAc → thalamus, and one connection from the hippocampus → thalamus revealed an interaction between study condition and stimulation effects.

**Table 2:** Results from the analysis of variance (ANOVA) comparing the effects of stimulation with the study condition (Condition X Stimulation).

**ANCOVA-Condition X Pain Score**					
	**Source**	**Target**	***p*** **-value**	**Source Sub-Region**	**Target Sub-Region**
**Stimulation**					
	Thalamus	IC	3.09 ×10^−8^	4	6
	Thalamus	IC	3.72 ×10^−7^	4	1
	Thalamus	IC	6.01 ×10^−7^	4	2
	Thalamus	IC	1.86 ×10^−6^	4	3
	Thalamus	IC	2.95 ×10–^6^	5	3
	Thalamus	IC	4.68 ×10^−6^	4	4
	Amygdala	Hippocampus	4.90 ×10^−6^	4	1
	Thalamus	IC	8.91 ×10^−6^	4	5
	ACC	IC	9.77 ×10^−6^	7	1
	Thalamus	IC	1.07 ×10^−5^	5	6
	Thalamus	IC	1.48 ×10^−5^	4	7
	IC	Auditory	1.55 ×10^−5^	4	7
	Thalamus	IC	1.78 ×10^−5^	5	2
	Thalamus	IC	1.82 ×10^−5^	7	3
	IC	Amygdala	1.91 ×10–^5^	6	4
	IC	Auditory	2.04 ×10^−5^	1	7
**Condition**	NAc	Thalamus	1.45 ×10^−5^	4	3
**Interaction**	Hippocampus	Thalamus	9.33 ×10^−6^	7	3

*Source indicates the modelled region providing input signalling to a modelled target region. The sub-region number indicates specific sub-regions out of seven for each region, which were identified by the ANOVA to have significant changes in connectivity based on Stimulation, Condition, or an Interaction*.

**Figure 5:**
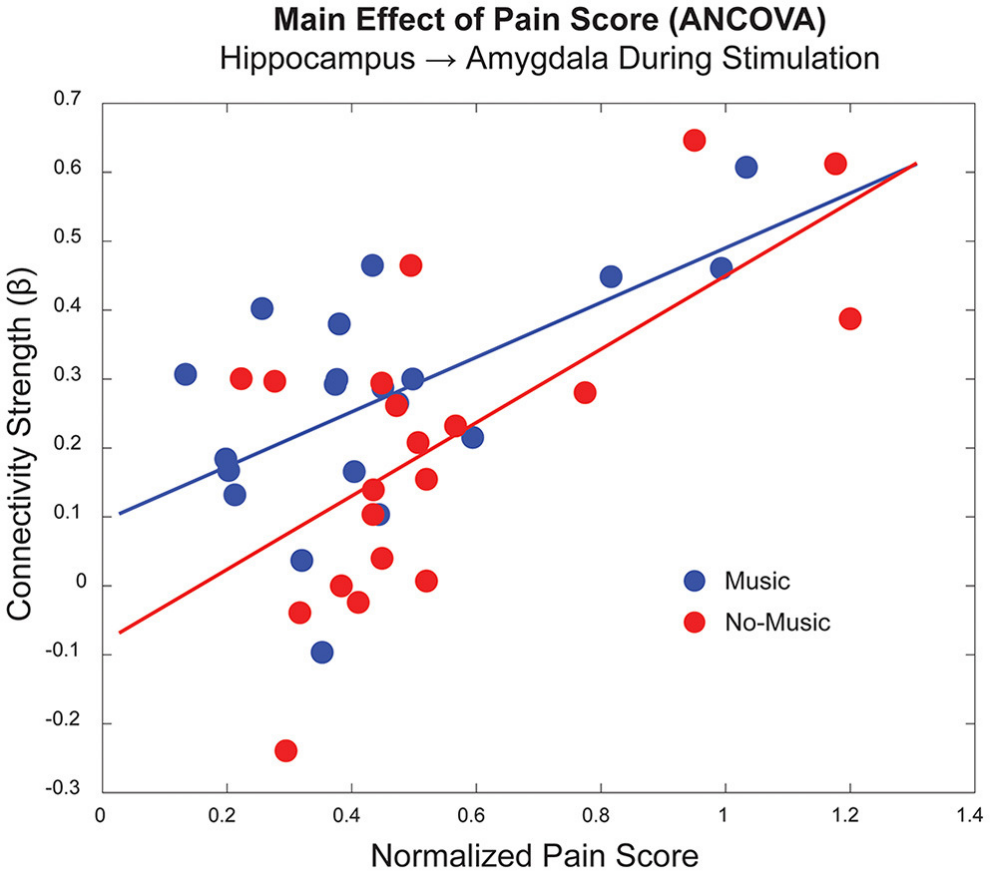
Example of a main effect of pain score for a connection between the hippocampus and amygdala in the period during noxious stimulation, as shown in Table 3 (*p* < 1.99 x 10^−5^). The horizontal axis indicates the average normalised pain score for each participant/condition, and the vertical axis indicates the connectivity strength (β) for this particular connection. The Music condition is displayed in blue, and the No-Music condition in red.

An ANCOVA was used to investigate the relationship between the study condition and individual pain sensitivity using the normalised pain scores (Condition X Pain Score). A widespread set of connections across the brain and brainstem demonstrated significant main effects of Pain Scores and Condition, as well as one significant Interaction effect (Table 3). The ANCOVA identified significant effects of pain scores before and during noxious stimulation from the PCC → thalamus and the hippocampus → amygdala, respectively. An respectively. An example of this effect is shown in Figure 5, indicating a positive relationship between individual pain scores and connectivity strengths (β) for the hippocampus → amygdala connection during the experience of pain. The significant main effects of the study condition in this comparison were driven mainly by connections involving the hippocampus and thalamus. More specifically, we identified the following connections that differed across study conditions: **before stimulation**, hypothalamus → LC and NAc → amygdala; **during stimulation**, hippocampus → thalamus and insula → amygdala; **after stimulation**, PCC → thalamus, hippocampus → amygdala, and auditory cortex → insula. Only one connection from the PAG → thalamus was identified to have significant interaction effects in the period after stimulation.

**Table 3:** Results from the analysis of covariance (ANCOVA) comparing individual pain scores to the study condition (Condition X Pain Score) at all time periods of the paradigm (before, during, and after stimulation). Source indicates the modelled region providing input signalling to a modelled target region.

**ANCOVA-Condition X Pain Score**						
		**Source**	**Target**	***p*** **-value**	**Source Sub-Region**	**Target Sub-Region**
Main effect of pain score	Before stim	PCC	Thalamus	1.82 ×10^−5^	1	3
	During stim	Hippocampus	Amygdala	1.99 ×10^−5^	5	4
	After stim	-	-	-	-	-
Main effect of Study condition	Before stim	Hypothalamus	LC	4.17 ×10^−6^	4	5
		NAc	Thalamus	5.62 ×10^−6^	4	3
	During stim	Hippocampus	Thalamus	4.37 ×10^−6^	7	7
		Hippocampus	Thalamus	8.32 ×10^−6^	7	3
		IC	Amygdala	2.04 ×10^−5^	7	3
	After stim	PCC	Thalamus	3.98 ×10^−6^	7	5
		Hippocampus	Amygdala	1.55 ×10^−5^	3	6
		Auditory	IC	2.14 ×10^−5^	6	1
Interaction effect	Before stim	-	-	-	-	-
	During stim	-	-	-	-	-
	After stim	PAG	Thalamus	7.76 ×10^−6^	6	7

*The sub-region number indicates specific sub-regions out of seven for each region, which were identified by the ANCOVA to have significant changes in connectivity based on Pain Scores, Condition, or Interaction effects*.

### Bayesian Regression Results

**Figure 6:**
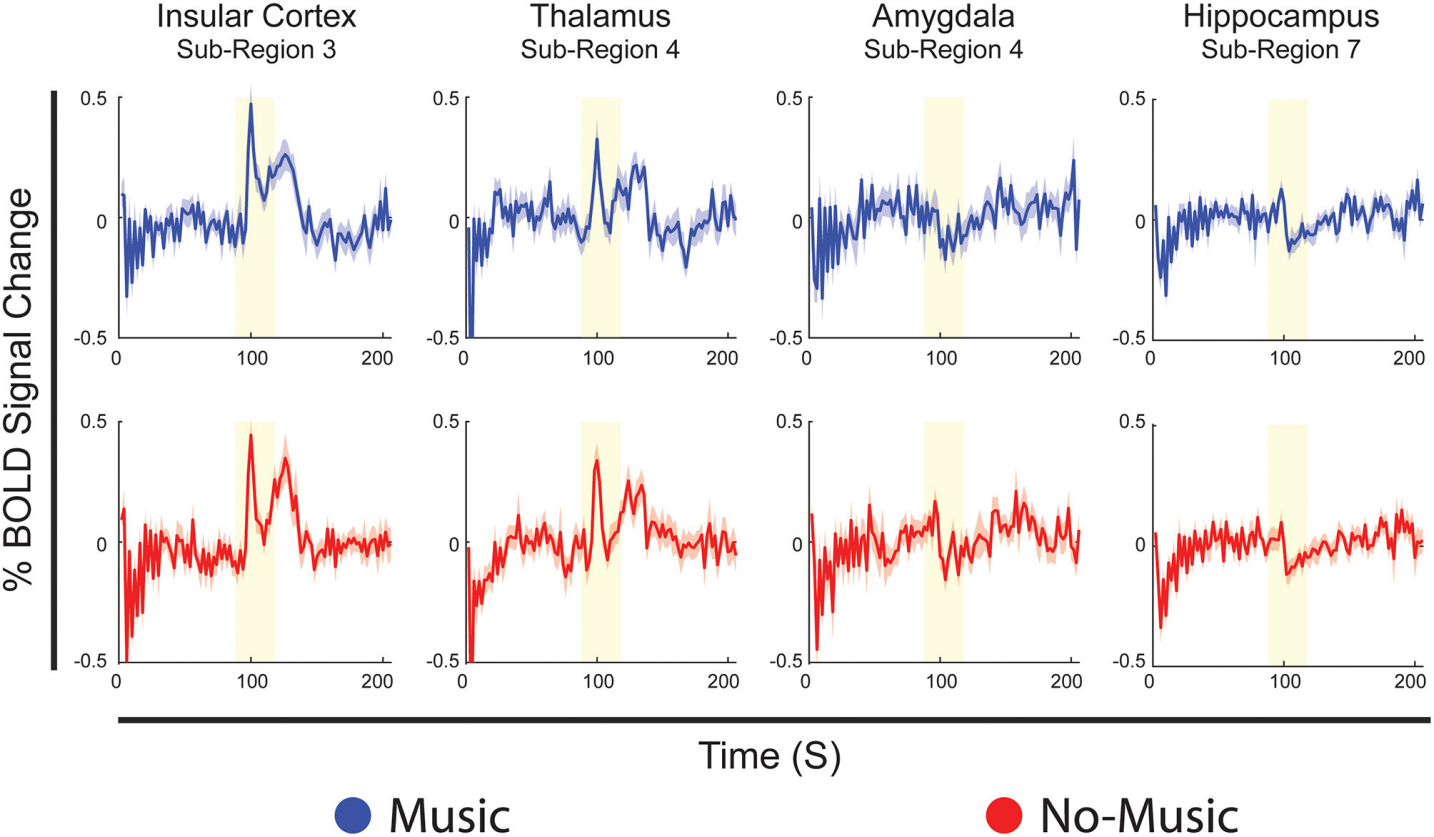
Examples of Bayesian regression results showing average BOLD time-courses from specific sub-regions, identical for both conditions, selected from the analyses of variance and covariance. Time-courses are displayed in blue for the Music condition, and red for the No-Music condition. The vertical axis indicates percent BOLD signal change from the mean and the horizontal axis indicates time in seconds. The vertical yellow bar indicates the period of noxious stimulation.

The results of the Bayesian regression analysis provided average time-courses for all sub-regions at the median pain rating and temperature used. Here, we provide examples of average time-courses in the Music and No-Music conditions from specific sub-regions, as identified by the ANOVA and ANCOVA analyses (Figure 6). We chose to show these particular regions as they are involved in both affective and discriminatory aspects of pain, and they clearly indicate reactive and continuous neural activity in response to different periods of the stimulation paradigm. Details of BOLD responses for all ROIs and sub-regions in the Music and No-Music conditions can be found in Supplementary Figures 1, 2.

## Discussion

This investigation provided evidence for behavioural and neural effects of music on the experience of pain in healthy individuals using functional MRI and showed that music affects pain regulation networks in specific ways. Compared with a No-Music condition, participants rated their pain unpleasantness significantly lower during the Music condition. This was reflected in significant network connectivity differences across conditions, in relation to normalised pain scores and the stimulus. Clear trends of cortico-limbic involvement in the effects of music reinforce the notion that music integrates cognitive, behavioural, emotional, and autonomic signalling to alter our perception of pain.

Although participants rated their pain unpleasantness 14% lower on average during the Music condition, their pain intensity scores, however, did not differ significantly across conditions. This is consistent with past behavioural studies of music analgesia which showed a decrease in unpleasantness but not intensity (10, 11), or a larger decrease in unpleasantness compared to intensity (4, 6), which could indicate that music analgesia involves more cognitive and affective modulation strategies than sensory/discriminative effects. Music has also been shown to significantly decrease pain intensity alone (5, 7, 37), however these studies did not include measures of pain unpleasantness. A recent review rejects the focus on reduction of pain intensity as a one-dimensional assessment of the pain experience, as it fails to reflect emotional and cognitive dimensions included in the contemporary holistic clinical approach of pain management (55). Cognitive and emotional pain modulation strategies may arise from familiarity, reward, and positive emotional valences that each participant attributed to their selections of music (i.e., happy, stimulating, etc.), leading to passive distraction from the acute experimental pain, as previously suggested (6, 18).

Significant relationships were found between pain unpleasantness scores and depression, and normalised pain scores and depression, however these measures are related via the stimulation temperature. Although no other relationships were found, it has been previously shown that personal characteristics including emotional and cognitive state, pain catastrophizing, autonomic symptoms, and familiarity with music significantly impact the pain experience, however a larger sample size is required to elucidate these behavioural relationships (6, 12, 18, 80–83). Additionally, a range of scores were recorded for each questionnaire, but most responders fell in the “normal” or average range, therefore no meaningful correlations could be established.

Analyses of variance identified specific differences in connectivity, as calculated by SEM, which were dependent on changes across study conditions and time periods within the stimulation paradigm. Main effects of noxious stimulation dominate the comparison, specifically differences between periods before and during noxious stimulation, indicating that stimulation itself produces larger effects on connectivity than music. Multiple connections between the thalamus and insula differed in strength between these periods, which may indicate strong reactive responses to pain in these regions. The thalamus is an important integration centre for afferent sensory input, and it relays noxious information to the posterior insular cortex which, in turn, acts as an integration point for nociception, emotion, salience, interoception, and autonomic homeostatic information (56, 57, 84, 85). This effect is also seen in results of the Bayesian regression (Figure 6), which demonstrate primarily reactive BOLD responses to noxious stimulation in both conditions in the insula and thalamus.

An effect of music was seen in all periods of the stimulation paradigm, compared with fewer effects of pain scores, and one interaction, calculated via analysis of covariance. Interestingly, insular connectivity that was affected by the study condition occurred only in the periods during and after stimulation, echoing the reactive, salient, effect of stimulation seen in ANOVA and Bayesian regression results in the insula. The involvement of the insula in the period after stimulation also supports evidence for integration of affective, cognitive, homeostatic, and interoceptive function, as participants experienced lingering after-sensations from noxious stimulation during this period and had opportunities to reflect on and appraise the pain that they had just felt (37, 56, 86). Affective processing surrounding the pain experience can also be inferred from this insular connection to the auditory cortex due to previous evidence for IC responses to emotional contents of auditory stimuli (87). Furthermore, music impacted connections between insula → amygdala and hippocampus → thalamus during the experience of pain, highlighting integration of limbic input in the effect of music analgesia of music analgesia (37, 88).

The ANCOVA also demonstrated a main effect of pain scores in two connections in the periods before and during noxious stimulation, indicating a potential priming effect of individuals' pain history and sensitivity on anticipation and sensation of pain. In the period before stimulation, participants experienced predictable anticipation of the impending pain, using this time for any natural behaviours including internally directed thought, daydreaming, expectation, etc. This effect may be inferred from a connection prior to stimulation between the PCC and thalamus, regions involved in the default mode network which is implicated in internally directed thought (89, 90). While the broad functions of the PCC are debated, it has been associated with emotional salience, discriminative avoidance learning, planning, attention, and episodic memory (90–92). The PCC and thalamus are both densely connected to limbic and paralimbic structures, including the amygdala and hippocampus, further implicating cognitive and emotional integration strategies in pain modulation (89, 93). The relationship with individual pain scores reinforces this suggestion as they relate to individual differences, memory, and cognitive/emotional appraisal of pain. Differences in cognitive strategies for pain modulation have been shown to be mediated by communication between regions involved in executive control and those involved with the “salience network” which includes many limbic regions (85, 94). Interestingly, connectivity between the amygdala and the hippocampus, largely involved with learning, memory, and emotion (95), varies based on pain scores during the period of noxious stimulation. This connection may further demonstrate the effects of personal pain history and sensitivity on the cognitive/emotional context during the subjective experience of pain. The strong relationship between pain behaviours and neural activity can be seen in the plot of this connection between these two regions during noxious stimulation (Figure 5).

Bayesian regression analyses demonstrate temporal properties of BOLD responses and show predictable, reactive responses to noxious stimulation in regions such as the insula and thalamus, indicating predominantly sensory/discriminative signalling effects. Regions such as the amygdala and hippocampus show more continuous signalling, suggesting potential cognitive/affective integration across the paradigm (Figure 6). While reactive responses to the stimulus are quite similar across conditions in the insula and thalamus, the amygdala and hippocampus show greater changes in signal amplitude across conditions during stimulation. This further reinforces the notion that limbic regions may work to modulate our perception of pain as we anticipate, experience, and recover from it, rather than simply reacting to a noxious sensation. Noticeable differences in BOLD signal fluctuations across Music and No-Music conditions are seen in the periods before and after stimulation in all regions, indicating altered anticipation and relief across conditions. Lastly, regions such as the insula, frontal cortex, and ACC reacted most strongly to a change in the period of the stimulation paradigm (i.e., onset of scanning, onset/offset of pain), suggesting that salience to a change in our environment plays a role in the holistic experience of pain (Supplementary Figures 1, 2) (96, 97).

Although this study demonstrated important broad effects of music analgesia across neural networks in the brain and brainstem, there are limitations to consider. While there is a wealth of behavioural knowledge regarding music analgesia, there is limited functional neurological data to build upon. Functional MRI is an inherently indirect method and, as such, provides information about neural activity via changes in blood oxygenation, which are related to the local metabolic demand. However, we do not have information regarding excitatory or inhibitory signalling. Additionally, the noise of the scanner may compete with the sound of the music, potentially confounding the analgesic effects. SEM is based on a pre-determined anatomical model and therefore contains limited information, for example some possible anatomical connections were omitted to decrease the number of multiple comparisons and necessary computing power. Even so, we were limited to describing the main findings related to the hypothesis, as these analyses produce too many detailed results to discuss in one text. Additionally, to maximise data quality in small brainstem regions, our field of view omitted superior regions of the cortex and therefore we could not capture the primary somatosensory cortex, which is directly involved in the sensory experience of pain. The fMRI methods were optimised for brain regions, and challenges with imaging in the lower brainstem regions may also have limited BOLD sensitivity in these regions. Our goal when calibrating the stimulation temperature is to produce the same approximate pain intensity (i.e., moderate pain) in all participants. As seen in Figure 5, the individual differences in normalised pain scores (pain unpleasantness rating / temperature °C) are closely related to the connectivity values seen across participants and conditions. Despite individual variability across participants we were still able to detect significant differences in network connectivity between Music and No-Music conditions, providing evidence for a neural basis of music analgesia. Additional investigations should be undertaken in the future to specifically address individual differences in functional data of this type and extend the age range beyond young adults. Finally, it is difficult to distinguish effects of cognition, emotion, salience, attention/distraction, and expectation of treatment (music), as these are closely linked. None the less, we believe that our results accurately reflect the complex network of interconnected regions with many functions that contribute to the pain experience (98).

Here, we have provided evidence for the behavioural and neural effects of music analgesia through individual ratings of pain, and changes in network connectivity by means of fMRI. The effect of music on pain perception appears to involve cognition, emotion, memory, salience, and multi-sensory integration, and serves to reduce primarily the unpleasantness of pain. Connecting with music on an emotional level may have the advantage of reducing pain in predictable scenarios such as medical procedures and positively impact the quality of life and daily function of those living with chronic pain.

## Data Availability Statement

The raw data supporting the conclusions of this article will be made available by the authors, without undue reservation.

## Ethics Statement

The studies involving human participants were reviewed and approved by Queen's University Health Sciences and Affiliated Teaching Hospitals Research Ethics Board. The patients/participants provided their written informed consent to participate in this study.

## Author Contributions

PS and JP designed the study and carried out data analysis. PS, JP, and GI carried out data collection. All authors have read and approved of the paper. All authors contributed to interpretation of the results and writing of the manuscript.

## Funding

This work was supported by the Natural Sciences and Engineering Research Council of Canada (NSERC) (Grant Number RGPIN/06221-2015).

## Conflict of Interest

The authors declare that the research was conducted in the absence of any commercial or financial relationships that could be construed as a potential conflict of interest.

## Publisher's Note

All claims expressed in this article are solely those of the authors and do not necessarily represent those of their affiliated organizations, or those of the publisher, the editors and the reviewers. Any product that may be evaluated in this article, or claim that may be made by its manufacturer, is not guaranteed or endorsed by the publisher.

## References

[1] Cepeda MS Carr DB Lau J Alvarez H. Music for pain relief. Cochrane Database Syst Rev. (2006) 2:CD004843. 10.1002/14651858.CD004843.pub2 16625614

[2] Lu X Yi F Hu L. Music-induced analgesia: an adjunct to pain management. Psychol Music. (2021) 49:1165–78. 10.1177/030573562092858531853196

[3] Lunde SJ Vuust P Garza-Villarreal EA Vase L. Music-induced analgesia: how does music relieve pain? Pain. (2019) 160:989–93. 10.1097/j.pain.0000000000001452 30507782

[4] Hsieh C Kong J Kirsch I Edwards RR Jensen KB Kaptchuk TJ. Well-loved music robustly relieves pain: a randomized, controlled trial. PLoS ONE. (2014) 9:e107390. 10.1371/journal.pone.0107390 25211164 PMC4161415

[5] Mitchell LA MacDonald RA. An experimental investigation of the effects of preferred and relaxing music listening on pain perception. J Music Ther. (2006) 43:295–316. 10.1093/jmt/43.4.295 17348757

[6] Garza-Villarreal EA Wilson AD Vase L Brattico E Barrios FA Jensen TS. Music reduces pain and increases functional mobility in fibromyalgia. Front Psychol. (2014) 5:90. 10.3389/fpsyg.2014.00090 24575066 PMC3920463

[7] Perlini AH Viita KA. Audioanalgesia in the control of experimental pain. Can J Behav Sci. (1996) 28:292–301. 10.1037/0008-400X.28.4.292

[8] Pando-Naude V Barrios FA Alcauter S Pasaye EH Vase L Brattico E. Functional connectivity of music-induced analgesia in fibromyalgia. Sci Rep. (2019) 9:15486. 10.1038/s41598-019-51990-4 31664132 PMC6820536

[9] Cabon MLF-B A Genestet S Quinio B Misery L Woda A Bodéré C. Impact of music on first pain and temporal summation of second pain: a psychophysical pilot study. Music Percept. (2021) 38:267–81. 10.1525/mp.2021.38.3.267

[10] Lu X Thompson WF Zhang L Hu L. Music reduces pain unpleasantness: evidence from an EEG study. J Pain Res. (2019) 12:3331–42. 10.2147/JPR.S212080 31853196 PMC6916681

[11] Garcia RL Hand CJ. Analgesic effects of self-chosen music type on cold pressor-induced pain: motivating vs. relaxing music. Psychol Music. (2016) 44:967–83. 10.1177/0305735615602144

[12] Chai PR Gale JY Patton ME Schwartz E Jambaulikar GD Wade Taylor S. The impact of music on nociceptive processing. Pain Med. (2020) 21:3047–54. 10.1093/pm/pnaa070 32337605 PMC7685689

[13] Antioch I Furuta T Uchikawa R Okumura M Otogoto J Kondo E. Favorite music mediates pain-related responses in the anterior cingulate cortex and skin pain thresholds. J Pain Res. (2020) 13:2729–37. 10.2147/JPR.S276274 33154663 PMC7605953

[14] Furuta T. The effects of auditory stimulation with pleasant and unpleasant sound on the pain threshold of gingiva and skin. Oral Health Dent Sci. (2019) 3:1–5. 10.33425/2639-9490.1044

[15] Lee JH. The effects of music on pain: a meta-analysis. J Music Ther. (2016) 53:430–77. 10.1093/jmt/thw012 27760797

[16] Hauck M Metzner S Rohlffs F Lorenz J Engel AK. The influence of music and music therapy on pain-induced neuronal oscillations measured by magnetencephalography. Pain. (2013) 154:539–47. 10.1016/j.pain.2012.12.016 23414577

[17] Mitchell LA MacDonald RA Brodie EE. A comparison of the effects of preferred music, arithmetic and humor on cold pressor pain. Eur J Pain. (2006) 10:343–51. 10.1016/j.ejpain.2005.03.005 15878297

[18] Pereira CS Teixeira J Figueiredo P Xavier J Castro SL Brattico E. Music and emotions in the brain: familiarity matters. PLoS ONE. (2011) 6:e27241. 10.1371/journal.pone.0027241 22110619 PMC3217963

[19] Villemure C Bushnell MC. Cognitive modulation of pain: how do attention and emotion influence pain processing? Pain. (2002) 95:195–9. 10.1016/S0304-3959(02)00007-6 11839418

[20] Vuilleumier P. How brains beware: neural mechanisms of emotional attention. Trends Cogn Sci. (2005) 9:585–94. 10.1016/j.tics.2005.10.011 16289871

[21] Tracey I Mantyh PW. The cerebral signature for pain perception and its modulation. Neuron. (2007) 55:377–91. 10.1016/j.neuron.2007.07.012 17678852

[22] Li X Hu L. The role of stress regulation on neural plasticity in pain chronification. Neural Plast. (2016) 2016:6402942. 10.1155/2016/6402942 28053788 PMC5178373

[23] McKinney CH Antoni MH Kumar M Tims FC McCabe PM. Effects of guided imagery and music (GIM) therapy on mood and cortisol in healthy adults. Health Psychol. (1997) 16:390–400. 10.1037/0278-6133.16.4.390 9237092

[24] McKinney CH Tims FC Kumar AM Kumar M. The effect of selected classical music and spontaneous imagery on plasma beta-endorphin. J Behav Med. (1997) 20:85–99. 10.1023/A:1025543330939 9058181

[25] Salimpoor VN Benovoy M Larcher K Dagher A Zatorre RJ. Anatomically distinct dopamine release during anticipation and experience of peak emotion to music. Nat Neurosci. (2011) 14:257–62. 10.1038/nn.2726 21217764

[26] Chai PR Carreiro S Ranney ML Karanam K Ahtisaari M Edwards R. Music as an adjunct to opioid-based analgesia. J Med Toxicol. (2017) 13:249–54. 10.1007/s13181-017-0621-9 28646359 PMC5570730

[27] Stefano GB Zhu W Cadet P Salamon E Mantione KJ. Music alters constitutively expressed opiate and cytokine processes in listeners. Med Sci Monit. (2004) 10:MS18–27. 15173680

[28] Blood AJ Zatorre RJ. Intensely pleasurable responses to music correlate with activity in brain regions implicated in reward and emotion. Proc Natl Acad Sci U S A. (2001) 98:11818–23. 10.1073/pnas.191355898 11573015 PMC58814

[29] Wise RA. Dopamine, learning and motivation. Nat Rev Neurosci. (2004) 5:483–94. 10.1038/nrn1406 15152198

[30] Salimpoor VN Zald DH Zatorre RJ Dagher A McIntosh AR. Predictions and the brain: how musical sounds become rewarding. Trends Cogn Sci. (2015) 19:86–91. 10.1016/j.tics.2014.12.001 25534332

[31] Zatorre RJ. Musical pleasure and reward: mechanisms and dysfunction. Ann N Y Acad Sci. (2015) 1337:202–11. 10.1111/nyas.12677 25773636

[32] Tu Y Bi Y Zhang L Wei H Hu L. Mesocorticolimbic pathways encode cue-based expectancy effects on pain. J Neurosci. (2020) 40:382–94. 10.1523/JNEUROSCI.1082-19.2019 31694965 PMC6948945

[33] Ploghaus A Narain C Beckmann CF Clare S Bantick S Wise R. Exacerbation of pain by anxiety is associated with activity in a hippocampal network. J Neurosci. (2001) 21:9896–903. 10.1523/JNEUROSCI.21-24-09896.2001 11739597 PMC6763058

[34] Reynolds DV. Surgery in the rat during electrical analgesia induced by focal brain stimulation. Science. (1969) 164:444–5. 10.1126/science.164.3878.444 4887743

[35] Fields HL Basbaum AI. Central nervous system mechanisms of pain modulation. In: Wall PD Melzack R, editors. Textbook of Pain. New York, NY: Churchill Livingstone (1994). p. 243–57.

[36] Potvin S Grignon S Marchand S. Human evidence of a supra-spinal modulating role of dopamine on pain perception. Synapse. (2009) 63:390–402. 10.1002/syn.20616 19173266

[37] Dobek CE Beynon ME Bosma RL Stroman PW. Music modulation of pain perception and pain-related activity in the brain, brain stem, and spinal cord: a functional magnetic resonance imaging study. J Pain. (2014) 15:1057–68. 10.1016/j.jpain.2014.07.006 25077425

[38] DunbarRIKaskatisKMacDonaldIBarraV. Performance of music elevates pain threshold and positive affect: implications for the evolutionary function of music. Evol Psychol. (2012) 10:688–702. 10.1177/14747049120100040323089077

[39] Garza-Villarreal EA Jiang Z Vuust P Alcauter S Vase L Pasaye EH. Music reduces pain and increases resting state fMRI BOLD signal amplitude in the left angular gyrus in fibromyalgia patients. Front Psychol. (2015) 6:1051. 10.3389/fpsyg.2015.01051 26257695 PMC4510313

[40] Roy M Lebuis A Hugueville L Peretz I Rainville P. Spinal modulation of nociception by music. Eur J Pain. (2012) 16:870–7. 10.1002/j.1532-2149.2011.00030.x 22337476

[41] Beck AT Steer RA Ball R Ranieri W. Comparison of beck depression inventories -IA and -II in psychiatric outpatients. J Pers Assess. (1996) 67:588–97. 10.1207/s15327752jpa6703_13 8991972

[42] Spielberger CD. State-Trait Anxiety Inventory for Adults (STAI-AD). APA PsycTests. (1983) 10.1039/t06496-000

[43] Crowne DP Marlowe D. A new scale of social desirability independent of psychopathology. J Consult Psychol. (1960) 24:349–54. 10.1037/h0047358 13813058

[44] Sullivan MJ Bishop SR Pivik J. The pain catastrophizing scale: development and validation. Psychol Assess. (1995) 7:524. 10.1037/1040-3590.7.4.52428616005

[45] Garza-Villarreal EA Pando V Vuust P Parsons C. Music-induced analgesia in chronic pain conditions: a systematic review and meta-analysis. Pain Physician. (2017) 20:597–610. 10.36076/ppj/2017.7.597 29149141

[46] Vierck CJ. Jr. Cannon RL Fry G Maixner W Whitsel BL. Characteristics of temporal summation of second pain sensations elicited by brief contact of glabrous skin by a preheated thermode. J Neurophysiol. (1997) 78:992–1002. 10.1152/jn.1997.78.2.992 9307129

[47] Price DD Hu JW Dubner R Gracely RH. Peripheral suppression of first pain and central summation of second pain evoked by noxious heat pulses. Pain. (1977) 3:57–68. 10.1016/0304-3959(77)90035-5 876667

[48] Staud R Cannon RC Mauderli AP Robinson ME Price DD Vierck CJ Jr.. Temporal summation of pain from mechanical stimulation of muscle tissue in normal controls and subjects with fibromyalgia syndrome. Pain. (2003) 102:87–95. 10.1016/s0304-3959(02)00344-5 12620600

[49] Yessick LR Pukall CF Ioachim G Chamberlain SM Stroman PW. An investigation of descending pain modulation in women with Provoked Vestibulodynia (PVD): alterations of spinal cord and brainstem connectivity. Front Pain Res. (2021) 2:682483. 10.3389/fpain.2021.682483 35295532 PMC8915748

[50] Bosma RL Stroman PW. Spinal cord response to stepwise and block presentation of thermal stimuli: a functional MRI study. J Magn Reson Imaging. (2014) 41:1318–25. 10.1002/jmri.24656 24807470

[51] Stroman PW Powers JM Ioachim G Warren HJM McNeil K. Investigation of the neural basis of expectation-based analgesia in the human brainstem and spinal cord by means of functional magnetic resonance imaging. Neurobiol Pain. (2021) 10:100068. 10.1016/j.ynpai.2021.100068 34381928 PMC8333346

[52] Staud R Craggs JG Robinson ME Perlstein WM Price DD. Brain activity related to temporal summation of C-fiber evoked pain. Pain. (2007) 129:130–42. 10.1016/j.pain.2006.10.010 17156923 PMC1997296

[53] Staud R Robinson ME Price DD. Temporal summation of second pain and its maintenance are useful for characterizing widespread central sensitization of fibromyalgia patients. J Pain. (2007) 8:893–901. 10.1016/j.jpain.2007.06.006 17681887 PMC2174917

[54] Millan MJ. Descending control of pain. Prog Neurobiol. (2002) 66:355–474. 10.1016/S0301-0082(02)00009-6 12034378

[55] Porreca F Navratilova E. Reward, motivation, and emotion of pain and its relief. Pain. (2017) 158:S43–9. 10.1097/j.pain.0000000000000798 28106670 PMC5350036

[56] Craig AD. Interoception: the sense of the physiological condition of the body. Curr Opin Neurobiol. (2003) 13:500–5. 10.1016/S0959-4388(03)00090-4 12965300

[57] Craig AD. A new view of pain as a homeostatic emotion. Trends Neurosci. (2003) 26:303–7. 10.1016/S0166-2236(03)00123-1 12798599

[58] Warren HJM Ioachim G Powers JM Stroman PW. How fMRI -Fibromyalgia. J Pain Res. (2021) 14:381–98. 10.2147/JPR.S290795 33603453 PMC7882802

[59] Whitfield-Gabrieli S Nieto-Castanon A. Conn: a functional connectivity toolbox for correlated and anticorrelated brain networks. Brain Connect. (2012) 2:125–41. 10.1089/brain.2012.0073 22642651

[60] Liebe T Kaufmann J Li M Skalej M Wagner G Walter M. *In vivo* anatomical mapping of human locus coeruleus functional connectivity at 3 T MRI. Hum Brain Mapp. (2020) 41:2136–51. 10.1002/hbm.24935 31994319 PMC7267980

[61] Chiang MC Bowen A Schier LA Tupone D Uddin O Heinricher MM. Parabrachial complex: a hub for pain and aversion. J Neurosci. (2019) 39:8225–30. 10.1523/JNEUROSCI.1162-19.2019 31619491 PMC6794922

[62] Naidich TP Duvernoy HM Delman BN Sorensen AG Kollias SS Haacke EM. Internal Architecture of the Brain Stem With Key Axial Sections. Duvernoy's Atlas of the Human Brain Stem and Cerebellum. New York, NY: Springer-Verlag/Wien (2009). p. 79–82. 10.1007/978-3-211-73971-6

[63] Talairach J Tournoux P. Co-Planar Stereotaxic Atlas of the Human Brain. New York NY: Thieme Medical Publishers, Inc. (1988).

[64] Leijnse JN D'Herde K. Revisiting the segmental organization of the human spinal cord. J Anat. (2016) 229:384–93. 10.1111/joa.12493 27173936 PMC4974552

[65] Pauli WM Nili AN Tyszka JM. A high-resolution probabilistic *in vivo* atlas of human subcortical brain nuclei. Sci Data. (2018) 5:180063. 10.1038/sdata.2018.63 29664465 PMC5903366

[66] Keren NI Lozar CT Harris KC Morgan PS Eckert MA. *In vivo* mapping of the human locus coeruleus. Neuroimage. (2009) 47:1261–7. 10.1016/j.neuroimage.2009.06.012 19524044 PMC3671394

[67] Stroman PW. Validation of structural equation modeling methods for functional MRI data acquired in the human brainstem and spinal cord. Crit Rev Biomed Eng. (2016) 44:227–41. 10.1615/CritRevBiomedEng.2017020438 29199575

[68] Stroman PW Warren HJM Ioachim G Powers JM McNeil K. A comparison of the effectiveness of functional MRI analysis methods for pain research: the new normal. PLoS ONE. (2020) 15:e0243723. 10.1371/journal.pone.0243723 33315886 PMC7735591

[69] Bosma RL Ameli Mojarad E Leung L Pukall C Staud R Stroman PW. Neural correlates of temporal summation of second pain in the human brainstem and spinal cord. Hum Brain Mapp. (2015) 36:5038–50. 10.1002/hbm.22993 26366748 PMC6869075

[70] Khan HS Stroman PW. Inter-individual differences in pain processing investigated by functional magnetic resonance imaging of the brainstem and spinal cord. Neuroscience. (2015) 307:231–41. 10.1016/j.neuroscience.2015.08.059 26335379

[71] Bosma RL Mojarad EA Leung L Pukall C Staud R Stroman PW. FMRI of spinal and supra-spinal correlates of temporal pain summation in fibromyalgia patients. Hum Brain Mapp. (2016) 37:1349–60. 10.1002/hbm.23106 26749315 PMC4783193

[72] Craig AD. How do you feel? interoception*:* the sense of the physiological condition of the body nature reviews. Neuroscience. (2002) 3:655–66. 10.1038/nrn894 12154366

[73] Harita S Ioachim G Powers J Stroman PW. Investigation of resting-state BOLD networks in the human brainstem and spinal cord. Neuroscience. (2019) 404:71–81. 10.1016/j.neuroscience.2019.02.009 30776404

[74] Ioachim G Powers JM Stroman PW. Comparing coordinated networks across the brainstem and spinal cord in the resting state and altered cognitive state. Brain Connect. (2019) 9:415–24. 10.1089/brain.2018.0659 30909725

[75] Ioachim G Powers JM Warren HJM Stroman PW. Coordinated human brainstem and spinal cord networks during the expectation of pain have elements unique from resting-state effects. Brain Sci. (2020) 10:568. 10.3390/brainsci10090568 32824896 PMC7565010

[76] Stroman PW Ioachim G Powers JM Staud R Pukall C. Pain processing in the human brainstem and spinal cord before, during and after the application of noxious heat stimuli. Pain. (2018) 159:2012–20. 10.1080/24740527.2019.1591821 29905656

[77] Harita S Stroman PW. Confirmation of resting-state BOLD fluctuations in the human brainstem and spinal cord after identification and removal of physiological noise. Magn Reson Med. (2017) 78:2149–56. 10.1002/mrm.26606 28074492

[78] Stroman PW Ioachim G Powers JM Staud R Pukall C. Pain processing in the human brainstem and spinal cord before, during, and after the application of noxious heat stimuli. Pain. (2018) 159:2012–20. 29905656 10.1097/j.pain.0000000000001302

[79] Stroman PW Ioachim G Powers JM Staud R Pukall C. Pain processing in the human brainstem and spinal cord before, during, and after the application of noxious heat stimuli. Pain. (2018) 159:2012–20. 10.1097/j.pain.0000000000001302 29905656

[80] Kornelsen J McIver TA Stroman PW. Unique brain regions involved in positive versus negative emotional modulation of pain. Scand J Pain. (2019) 19:583–96. 10.1515/sjpain-2018-0341 31031262

[81] McIver TA Kornelsen J Stroman PW. Diversity in the emotional modulation of pain perception: an account of individual variability. Eur J Pain. (2018) 22:319–32. 10.1002/ejp.1122 28940720

[82] Tracey I Ploghaus A Gati JS Clare S Smith S Menon RS. Imaging attentional modulation of pain in the periaqueductal gray in humans. J Neurosci. (2002) 22:2748–52. 10.1523/JNEUROSCI.22-07-02748.2002 11923440 PMC6758341

[83] Villemure C Bushnell MC. Mood influences supraspinal pain processing separately from attention. J Neurosci. (2009) 29:705–15. 10.1523/JNEUROSCI.3822-08.2009 19158297 PMC2768393

[84] Lu C Yang T Zhao H Zhang M Meng F Fu H. Insular cortex is critical for the perception, modulation, and chronification of pain. Neurosci Bull. (2016) 32:191–201. 10.1007/s12264-016-0016-y 26898298 PMC5563738

[85] Seeley WW. The salience network: a neural system for perceiving and responding to homeostatic demands. J Neurosci. (2019) 39:9878–82. 10.1523/JNEUROSCI.1138-17.2019 31676604 PMC6978945

[86] Xue G Lu Z Levin IP Bechara A. The impact of prior risk experiences on subsequent risky decision-making: the role of the insula. Neuroimage. (2010) 50:709–16. 10.1016/j.neuroimage.2009.12.097 20045470 PMC2828040

[87] Zhang Y Zhou W Wang S Zhou Q Wang H Zhang B. The roles of subdivisions of human insula in emotion perception and auditory processing. Cereb Cortex. (2019) 29:517–28. 10.1093/cercor/bhx334 29342237

[88] Thompson JM Neugebauer V. Cortico-limbic pain mechanisms. Neurosci Lett. (2019) 702:15–23. 10.1016/j.neulet.2018.11.037 30503916 PMC6520155

[89] Leech R Sharp DJ. The role of the posterior cingulate cortex in cognition and disease. Brain. (2014) 137:12–32. 10.1093/brain/awt162 23869106 PMC3891440

[90] Brewer JA Garrison KA Whitfield-Gabrieli S. What about the “Self” is processed in the posterior cingulate cortex? Front Hum Neurosci. (2013) 7:647. 10.3389/fnhum.2013.00647 24106472 PMC3788347

[91] Maddock RJ Garrett AS Buonocore MH. Posterior cingulate cortex activation by emotional words: fMRI evidence from a valence decision task. Hum Brain Mapp. (2003) 18:30–41. 10.1002/hbm.10075 12454910 PMC6871991

[92] Maddock RJ Garrett AS Buonocore MH. Remembering familiar people: the posterior cingulate cortex and autobiographical memory retrieval. Neuroscience. (2001) 104:667–76. 10.1016/S0306-4522(01)00108-7 11440800

[93] Robertson RT Kaitz SS. Thalamic connections with limbic cortex. I thalamocortical projections J Comp Neurol. (1981) 195:501–25. 10.1002/cne.901950308 7204659

[94] Cheng JC Bosma RL Hemington KS Kucyi A Lindquist MA Davis KD. Slow-5 dynamic functional connectivity reflects the capacity to sustain cognitive performance during pain. Neuroimage. (2017) 157:61–8. 10.1016/j.neuroimage.2017.06.005 28583880

[95] Anand KS Dhikav V. Hippocampus in health and disease: an overview. Ann Indian Acad Neurol. (2012) 15:239–46. 10.4103/0972-2327.104323 23349586 PMC3548359

[96] Taylor KS Seminowicz DA Davis KD. Two systems of resting state connectivity between the insula and cingulate cortex. Hum Brain Mapp. (2009) 30:2731–45. 10.1002/hbm.20705 19072897 PMC6871122

[97] Legrain V Iannetti GD Plaghki L Mouraux A. The pain matrix reloaded: a salience detection system for the body. Prog Neurobiol. (2011) 93:111–24. 10.1016/j.pneurobio.2010.10.005 21040755

[98] Moayedi M Salomons TV Atlas LY. Pain neuroimaging in humans: a primer for beginners and non-imagers. J Pain. (2018) 19:e1–21. 10.1016/j.jpain.2018.03.011 29608974 PMC6192705

