# Supplementary material for: Music to My Senses: Functional Magnetic Resonance Imaging Evidence of Music Analgesia Across Connectivity Networks Spanning the Brain and Brainstem
Source: Front Pain Res (Lausanne). 2022 May 19;3:878258. doi: 10.3389/fpain.2022.878258 (PMC9160574; doi:10.3389/fpain.2022.878258)
Supplement: Supplementary file 1 [file Data_Sheet_1.pdf]

Supplementary Material

1 Supplementary Figures and Tables

1.1 Supplementary Figures

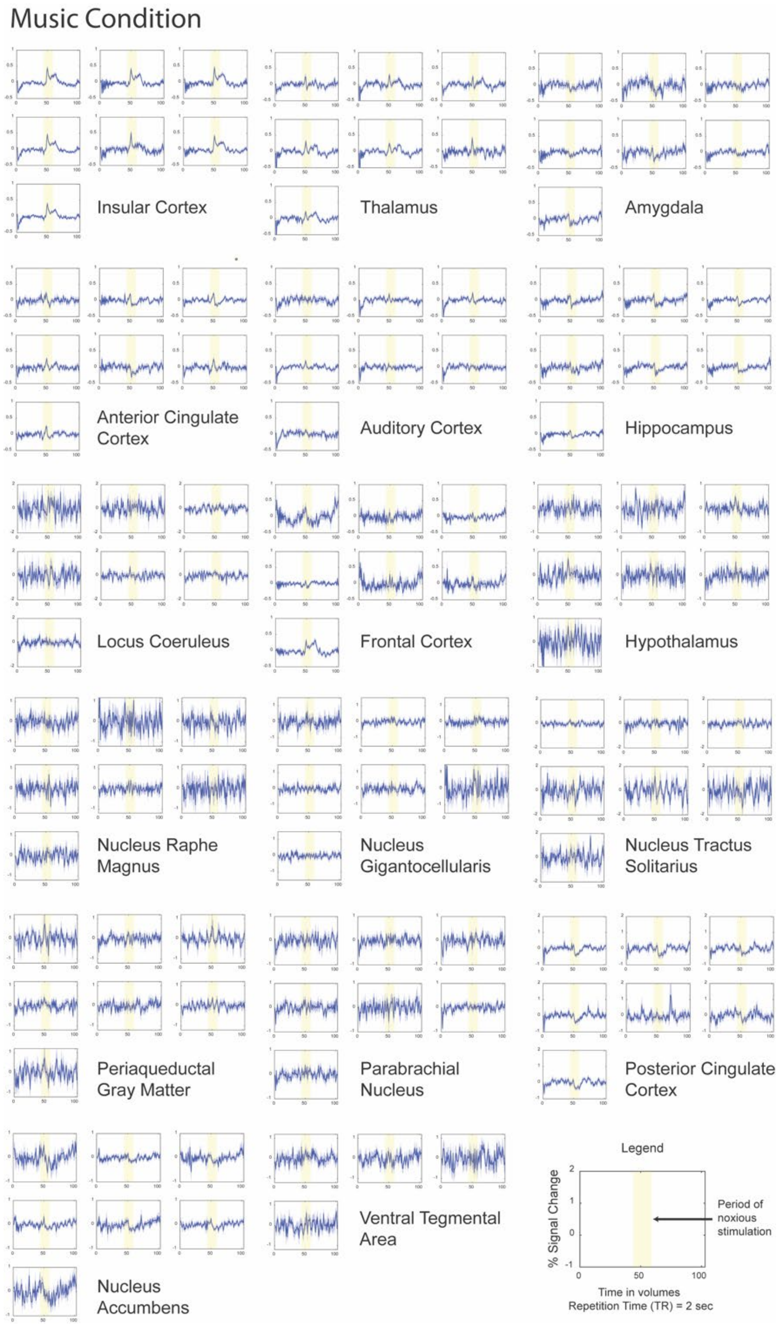

Supplementary Figure 1. Bayesian regression results, showing average BOLD time-courses for each sub-region during the Music condition. The vertical scale indicates percent BOLD signal change

from the mean and the horizontal scale indicates time in volumes of acquisition (TR = 2 sec). The vertical yellow bar indicates the period of noxious stimulation.

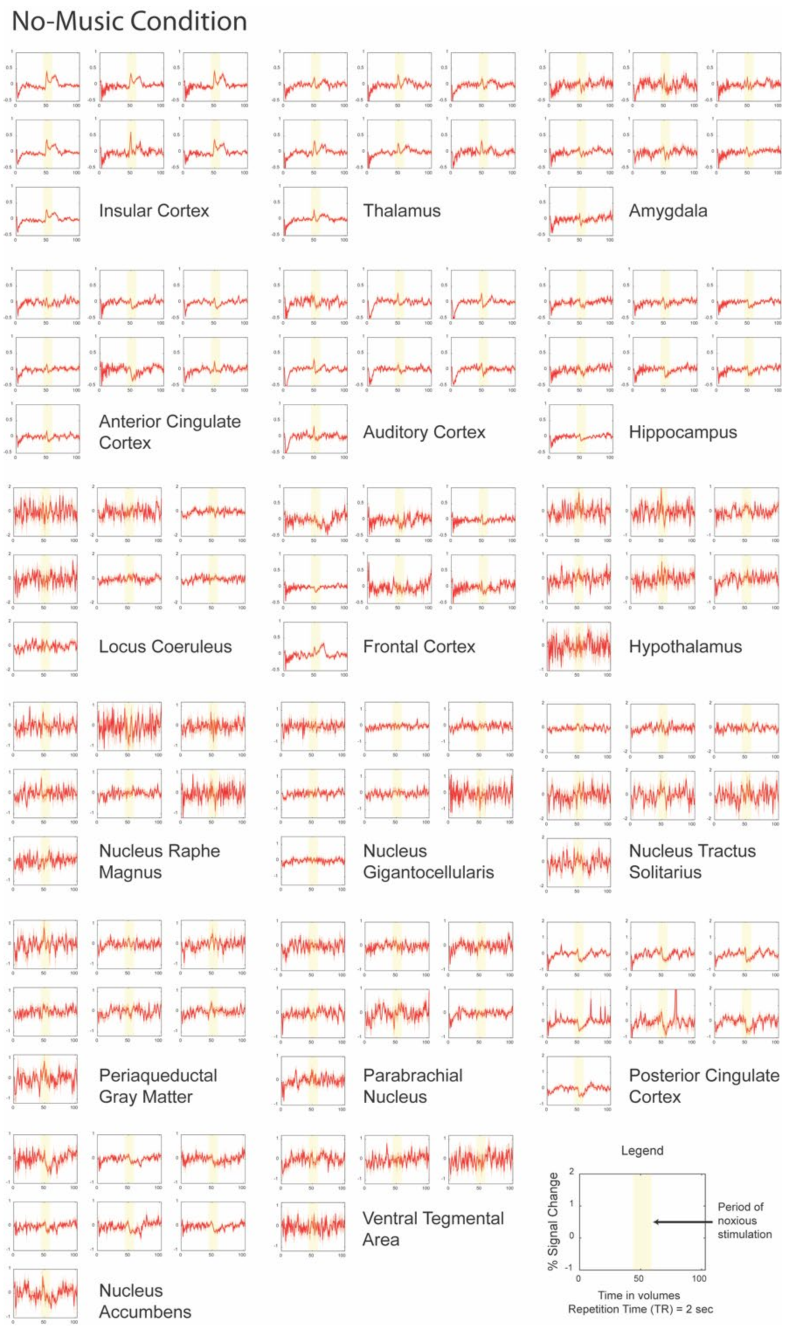

Supplementary Figure 2. Bayesian regression results, showing average BOLD time-courses for each ROI and sub-region during the No-Music condition. The vertical scale indicates percent BOLD signal change from the mean and the horizontal scale indicates time in volumes of acquisition (TR = 2 sec). The vertical yellow bar indicates the period of noxious stimulation.
